# Increased risk of incident gout in young men with metabolic syndrome: A nationwide population-based cohort study of 3.5 million men

**DOI:** 10.3389/fmed.2022.1010391

**Published:** 2022-11-14

**Authors:** Yeonghee Eun, Kyungdo Han, Seung Woo Lee, Kyunga Kim, Seonyoung Kang, Seulkee Lee, Hoon-Suk Cha, Eun-Mi Koh, Hyungjin Kim, Jaejoon Lee

**Affiliations:** ^1^Division of Rheumatology, Department of Internal Medicine, Kangbuk Samsung Hospital, Sungkyunkwan University School of Medicine, Seoul, South Korea; ^2^Department of Statistics and Actuarial Science, Soongsil University, Seoul, South Korea; ^3^Department of Biomedicine and Health Science, College of Medicine, The Catholic University of Korea, Seoul, South Korea; ^4^Statistics and Data Center, Research Institute for Future Medicine, Samsung Medical Center, Seoul, South Korea; ^5^Department of Digital Health, Samsung Advanced Institute for Health Sciences and Technology (SAIHST), Sungkyunkwan University, Seoul, South Korea; ^6^Department of Medicine, Samsung Medical Center, Sungkyunkwan University School of Medicine, Seoul, South Korea; ^7^Department of Medical Humanities, Samsung Medical Center, Sungkyunkwan University School of Medicine, Seoul, South Korea

**Keywords:** gout, metabolic syndrome, young men, crystal induced arthritis, risk factor

## Abstract

**Background:**

To date, few studies have focused on risk factors for gout in young people, and large-scale studies on the relationship between metabolic syndrome (MetS) and gout are lacking. We aimed to investigate the association between gout and MetS in a large nationwide population-based cohort of young men who participated in national health examination.

**Materials and methods:**

Cohort included men aged 20–39 years who participated in a health check-up in 2009–2012. A total of 3,569,104 subjects was included in the study, excluding those who had a previous diagnosis of gout or had renal impairment. The outcome was the occurrence of gout, which was defined using the diagnosis code of gout in the claims database. Cox proportional hazard model was used to evaluate the association between MetS and incident gout.

**Results:**

Mean follow-up duration was 7.35 ± 1.24 years and the incidence rate of gout was 3.36 per 1,000 person-years. The risk of gout in subjects with MetS was 2.4-fold higher than subjects without MetS. Among the components of MetS, hypertriglyceridemia and abdominal obesity showed the greatest association with gout. As the number of MetS components increased, the risk of gout increased. The association between gout and MetS was more pronounced in relatively young subjects and in low- or normal-weight subjects.

**Conclusion:**

Metabolic syndrome is an important risk factor for the gout in young men. In particular, the association between MetS and gout was greater in young and non-obese men. Management of MetS in young men will be important for future gout prevention.

## Introduction

Gout is the most common type of inflammatory arthritis in men, and the incidence has increased globally in recent decades (1). Although the incidence and prevalence rates of gout increase with age (2), the rate in young people is increasing. In South Korea, the prevalence of gout in individuals in their 20s and 30s increased by more than 4-fold in 2015 compared with that in 2002 (3). In addition, the age of onset of gout has decreasing (4, 5). Dietary risk factors for gout include alcohol intake, high-fructose sugar-sweetened drink consumption, and increasing daily servings of meat and seafood (2, 6). In addition, previous studies have reported that hypertension, hyperlipidemia, obesity, and renal disease are associated with a high risk of gout (7, 8). Genome-wide association studies identified genetic loci associated with uric acid handling to be associated with hyperuricemia and gout (9, 10).

Although the definition of early onset gout differs among studies, gout that occurs before the age of 30 or 40 generally is defined as early onset gout. Early onset gout has different characteristics from late-onset gout. In a Chinese 10-year observational study of 9,754 gout patients, those with early onset gout (onset age <30 years) had a higher body mass index (BMI) and less frequent alcohol drinking than those with late-onset gout (5). In a cross-sectional study of the French GOSPEL cohort, early onset gout (onset age <40 years) was associated with metabolic syndrome (MetS) (11). In a retrospective medical record review in the USA, patients with early onset gout (onset age <40 years) had higher BMI than those with late-onset gout. In that study, despite the young age of patients, the prevalence of hypertension and hyperlipidemia among patients with early onset gout was greater than 40% (12).

Metabolic syndrome is a cluster of cardiovascular risk factors, including hyperglycemia, elevated blood pressure (BP), elevated triglyceride (TG), low high-density lipoprotein (HDL) cholesterol, and central obesity (13). Growing evidence has indicated an association between gout and MetS. Previous studies revealed that the prevalence of MetS in individuals with gout was remarkably higher than in those without gout (14). A single-center study in South Korea showed higher prevalence of MetS in patients with gout than in the general Korean population (15). Hyperuricemia, the most important risk factor for gout, was associated with MetS, and the prevalence of hyperuricemia increased as the number of MetS components increased (16–18). In a study using data from the Korea National Health and Nutrition Examination Survey, the odds ratio for the association between hyperuricemia and MetS in men was 2.38 (95% confidence interval [CI] 1.84–3.08). Both gout and MetS are associated with activation of the renin-angiotensin system and insulin resistance, but the causal relationship between the two conditions has not been clarified (19).

To date, many studies have been conducted on risk factors for gout, but few have focused on risk factors in young people. In addition, studies have revealed a large correlation between gout and MetS in young people, but the data on this finding were obtained in a study performed in a cross-sectional manner with a small number of subjects. Therefore, we aimed to investigate risk factors for the occurrence of gout and the relationship between gout and MetS in a large nationwide population-based cohort of young men who participated in health examinations.

## Materials and methods

### Data resource

The Korean National Health Insurance Service (NHIS) is a government insurance provider that covers 97% of the population of South Korea. The remaining 3%, which belong to the lowest income bracket, is covered through another medical aid program also provided through NHIS. The NHIS database is a public database containing information on socio-demographic variables, healthcare utilization, health screening, and mortality of the population of South Korea (20, 21).

The NHIS offers a variety of health screening programs, including a cardiovascular health screening program every 2 years for all Koreans aged 40 years and older. The Ministry of Employment and Labor requires business owners to provide all employees with a general health examination that includes the same items as in the cardiovascular health screening program provided by NHIS. The participation rate of employees in their 20s and 30s in health checkup was 70–82% in 2009 and 77–84% in 2012 (22). The database linking the claims information of NHIS with the health examination data has been widely used in various epidemiological studies including in discovery of risk factors for diseases (23–25).

### Study population

Men aged 20–39 years who underwent a health check-up in 2009–2012 were included in the study to assess risk factors of gout and the effect of MetS on the risk of gout in young men. Exclusion criteria were kidney function impairment defined as estimated glomerular filtration rate (eGFR) <60 mL/min/1.73 m^2^ or albuminuria on urinalysis at the time of health examination, diagnosis of gout for 1 year before health examination, and one or more missing values for the variables of interest. Subjects who were diagnosed with gout or died within 1 year after the health examination date were excluded from the study.

The present study was conducted according to the guidelines of the Declaration of Helsinki and was approved by the Institutional Review Board of Samsung Medical Center (IRB File No. SMC 2021-10-008). The requirement for written informed consent was waived because this study was analyzed retrospectively using publicly available and anonymized data.

### Definition of metabolic syndrome

Metabolic syndrome was defined using criteria developed by the International Diabetes Federation; American Heart Association/National Heart, Lung, and Blood Institute; World Heart Federation; International Atherosclerosis Society; and International Association for the Study of Obesity (26). Subjects with three or more of the following risk factors were considered to have MetS: abdominal obesity (waist circumference ≥90 cm) (27); elevated TG (≥150 mg/dL or drug treatment for elevated TG); reduced HDL cholesterol (<40 mg/dL or drug treatment for reduced HDL cholesterol); elevated BP (systolic BP ≥130 mmHg and/or diastolic BP ≥85 mmHg or antihypertensive drug treatment in subjects with a history of hypertension); and elevated fasting glucose (≥100 mg/dL or drug treatment of elevated glucose). MetS status was assessed on the health check-up.

### Data collection

Supplementary Figure 1 shows the study design. The index date of cohort was defined as the date of the health check-up. Information on baseline comorbidities such as hypertension, diabetes mellitus, and hyperlipidemia was collected using diagnostic codes and information on prescribed medication for 1 year before the index date. Other variables of baseline characteristics were collected at the index date. Demographic variables such as age and sex were reviewed. Income was classified by quartile based on the premiums charged by the health insurance based on household income. As part of the health examination, body weight, height, waist circumference, and systolic and diastolic BP were measured. BMI was calculated by dividing body weight in kilograms by the square of height in meters and classified into five categories according to the Asian-Pacific cutoff points (28). Laboratory test items of the health examination included fasting glucose, total cholesterol, HDL cholesterol, low-density lipoprotein (LDL) cholesterol, TG, and creatinine. The Modification of Diet in Renal Disease formula was used for calculation of eGFR. The triglyceride glucose (TyG) index, a simple marker reflecting insulin resistance, was calculated as ln [TG (mg/dL) × fasting glucose (mg/dL)/2], based on previous studies (29, 30). Health-related behaviors such as smoking, alcohol, and regular exercise were evaluated through a self-reported questionnaire. Smoking status was classified as never, ex-, and current smoker. Alcohol drinking was classified into none (0 g/day), mild (<30 g/day), and heavy (≥30 g/day) drinking based on the amount of daily alcohol consumption. Regular exercise was defined as moderate physical activity for ≥30 min at least five times per week or vigorous physical activity for ≥20 min at least three times per week.

### Study outcomes and follow-up

The primary outcome of the study was incident gout, which was defined as three outpatient visits with the diagnosis code of gout [International classification of Disease 10th revision (ICD-10) code M10]. Cohort was followed from the index date until the outcome, death, or study end date (December 31, 2018), whichever came first.

### Statistical analysis

Continuous variables with normal distribution were expressed as mean ± standard deviation (SD), and categorical variables were expressed as number and percentage. The incidence rate of gout was calculated by dividing the number of incident cases by the total follow-up period [person-years (PYs)]. The Cox proportional hazard model was used to calculate hazard ratios (HRs) and 95% CIs for the risk of incident gout. The multivariable model was adjusted for age, sex, smoking, alcohol drinking, regular exercise, and income. The Kaplan-Meier survival curve was used to analyze the cumulative incidence of gout according to baseline MetS status. Adjusted cumulative incidence with the same variables as in the multivariable model was analyzed using Cox proportional hazard models. Stratified analysis was performed according to age and BMI subgroup. All statistical analyses were performed using SAS version 9.4 (SAS Institute Inc., Cary, NC, USA), and *p*-value less than 0.05 was considered statistically significant.

## Results

### Study population

**FIGURE 1:**
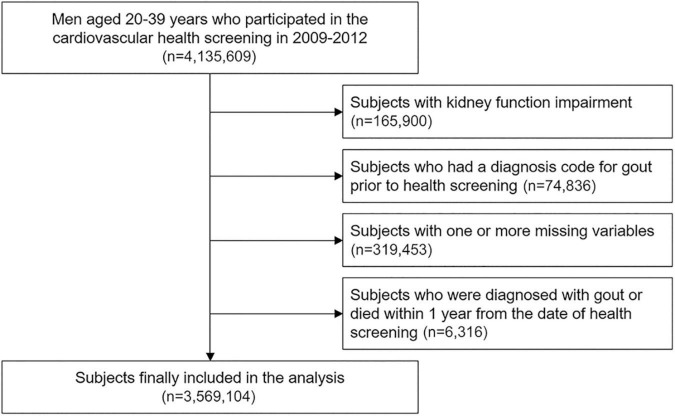
Flowchart of study population selection.

Among 4,135,609 men aged 20–39 years who participated in the health check-up in 2009–2012, 165,900 subjects with kidney function impairment and 74,836 subjects diagnosed with gout before the index date were excluded (Figure 1). After excluding 319,453 subjects with missing data and 6,316 subjects who were diagnosed with gout or died within 1 year after the index date, 3,569,104 subjects were included in cohort.

### Baseline characteristics

Table 1 presents baseline characteristics of study population. The mean age of study subjects was 31.5 ± 4.7 years. The percentage of subjects who had MetS at the time of medical examination were 14.6%. Subjects who had MetS at baseline were older, had a higher BMI and TyG index, and had higher rates of current smokers compared with subjects without MetS.

**TABLE 1:** Baseline characteristics of study population.

Variables	Total (*n* = 3,569,104)	Without MetS (*n* = 3,049,271)	With MetS (*n* = 519,833)	SMD
**Age**	31.5 ± 4.7	31.3 ± 4.7	33.2 ± 4.2	0.4371
20–29	1,293,621 (36.2)	1,181,933 (38.8)	111,688 (21.5)	0.3834
30–39	2,275,483 (63.8)	1,867,338 (61.2)	408,145 (78.5)	
**Income (quartile)**				0.0772
Q1 (lowest)	594,293 (16.7)	520,182 (17.1)	74,111 (14.3)	
Q2	1,118,021 (31.3)	967,677 (31.7)	150,344 (28.9)	
Q3	1,213,401 (34.0)	1,023,243 (33.6)	190,158 (36.6)	
Q4 (highest)	643,389 (18.0)	538,169 (17.7)	105,220 (20.2)	
**BMI (kg/m^2^)**	24.1 ± 3.4	23.5 ± 3.0	27.6 ± 3.5	1.2626
<18.5	96,700 (2.7)	95,800 (3.1)	900 (0.2)	0.2341
18.5–23	1,316,908 (36.9)	1,277,217 (41.9)	39,691 (7.6)	
23–25	871,734 (24.4)	799,453 (26.2)	72,281 (13.9)	
25–30	1,093,684 (30.6)	802,030 (26.3)	291,654 (56.1)	
≥30	190,078 (5.3)	74,771 (2.5)	115,307 (22.2)	
**Smoking**				0.1855
Never	1,087,843 (30.5)	966,054 (31.7)	121,789 (23.4)	
Ex-smoker	528,469 (14.8)	447,433 (14.7)	81,036 (15.6)	
Current smoker	1,952,792 (54.7)	1,635,784 (53.7)	317,008 (61.0)	
**Alcohol drinking**				0.0754
None	942,783 (26.4)	820,006 (26.9)	122,777 (23.6)	
Mild (<30 g/day)	2,157,163 (60.4)	1,854,668 (60.8)	302,495 (58.2)	
Heavy (≥30 g/day)	469,158 (13.1)	374,597 (12.3)	94,561 (18.2)	
Regular exercise	534,499 (15.0)	464,499 (15.2)	70,000 (13.5)	0.0504
**Comorbidities**				
Hypertension	372,403 (10.4)	207,699 (6.8)	164,704 (31.7)	0.6648
Diabetes mellitus	87,976 (2.5)	36,290 (1.2)	51,686 (9.9)	0.3889
Hyperlipidemia	310,954 (8.7)	196,184 (6.4)	114,770 (22.1)	0.4591
WC (cm)	82.1 ± 8.6	80.6 ± 7.5	91.1 ± 8.9	1.2789
Systolic BP (mmHg)	122.0 ± 12.3	120.3 ± 11.5	132.1 ± 12.2	0.9977
Diastolic BP (mmHg)	76.4 ± 9.1	75.2 ± 8.5	83.0 ± 9.5	0.8605
Fasting glucose (mg/dL)	92.5 ± 18.0	90.3 ± 13.8	105.3 ± 30.0	0.6416
Total C (mg/dL)	188.8 ± 37.0	186.0 ± 35.2	205.7 ± 42.7	0.5056
HDL C (mg/dL)	53.7 ± 25.7	55.0 ± 25.6	46.0 ± 25.3	0.3569
LDL C (mg/dL)	117.0 ± 229.6	116.7 ± 236.7	118.4 ± 182.1	0.0080
TG (mg/dL)^a^	117.9 (117.8–117.9)	105.9 (105.9–106.0)	220.2 (219.9–220.5)	1.4390
Creatinine (mg/dL)	1.01 ± 0.16	1.007 ± 0.157	1.008 ± 0.162	0.0118
eGFR (mL/min/1.73 m^2^)	96.9 ± 53.7	97.1 ± 54.4	95.6 ± 49.5	0.0304
**MetS**				
Abdominal obesity	621,278 (17.4)	291,532 (9.6)	329,746 (63.4)	1.3501
Elevated TG	1,183,402 (33.2)	722,714 (23.7)	460,688 (88.6)	1.7299
Reduced HDL C	451,560 (12.7)	223,095 (7.3)	228,465 (44.0)	0.9243
Elevated BP	1,273,111 (35.7)	856,224 (28.1)	416,887 (80.2)	1.2271
Elevated glucose	767,454 (21.5)	462,518 (15.2)	304,936 (58.7)	1.0096
**No of MetS components**				1.0804
0	1,209,361 (33.9)	1,209,361 (39.7)		
1	1,123,737 (31.5)	1,123,737 (36.9)		
2	716,173 (20.1)	716,173 (23.5)		
3	363,125 (10.2)		363,125 (69.9)	
4	132,193 (3.7)		132,193 (25.4)	
5	24,515 (0.7)		24,515 (4.7)	

Data are expressed as means ± SD, or *n* (%).

^a^Triglycerides were expressed as geometric means (95% confidence interval).

SMD, standardized mean difference; Q, quartile; BMI, body mass index; WC, waist circumference; BP, blood pressure; C, cholesterol; HDL, high-density lipoprotein; LDL, low-density lipoprotein; TG, triglycerides; eGFR, estimated glomerular filtration rate; MetS, metabolic syndrome; No, number.

### Association between baseline metabolic syndrome and gout

**FIGURE 2:**
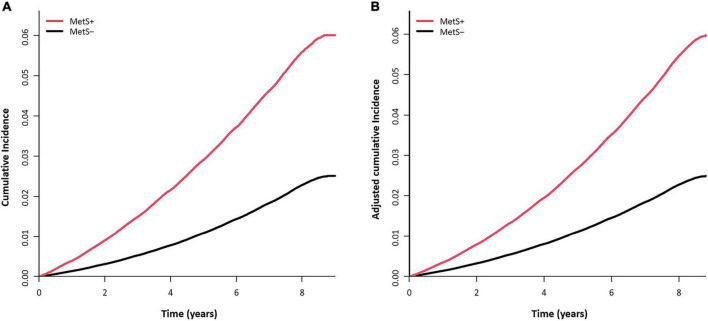
Cumulative incidence curve of gout according to baseline metabolic syndrome (MetS) status. **(A)** Unadjusted cumulative incidence probability and number of persons at risk for gout. **(B)** Adjusted cumulative incidence probability of gout and cumulative incidence (per 1,000 persons) at each time point. Adjusted for age, smoking, alcohol drinking, regular exercise, and income.

The mean follow-up duration was 7.4 ± 1.2 years. During the study period, 88,058 men were newly diagnosed with gout, and the incidence rate of gout was 3.36 per 1,000 PYs. The mean time to diagnosis of gout was 5.5 ± 2.2 years, and the mean age at gout diagnosis was 37.9 ± 5.0 years. MetS was associated with a more than doubled risk of gout [adjusted HR (aHR) 2.44, 95% CI 2.41–2.48; Table 2]. Among the components of MetS, abdominal obesity had the greatest association with gout (aHR 2.45, 95% CI 2.42–2.49), followed by elevated TG (aHR 2.29, 95% CI 2.26–2.32). The cumulative incidence of gout according to baseline MetS status is presented in Figure 2.

**TABLE 2:** Association between baseline metabolic syndrome status and incident gout.

	Subjects (*n*)	Events (*n*)	Follow-up duration (PYs)	Incidence rate (per 1,000 PYs)	Crude HR (95% CI)	aHR^a^ (95% CI)
Total	3,569,104	88,058	26,240,652	3.36		
**Metabolic syndrome**						
No	3,049,271	61,755	22,444,658	2.75	1 (Ref.)	1 (Ref.)
Yes	519,833	26,303	3,795,995	6.93	2.52 (2.48–2.55)	2.44 (2.41–2.48)
**Abdominal obesity**						
No	2,947,826	57,924	21,744,418	2.66	1 (Ref.)	1 (Ref.)
Yes	621,278	30,134	4,496,234	6.70	2.53 (2.50–2.57)	2.45 (2.42–2.49)
**Elevated TG**						
No	2,385,702	40,616	17,514,435	2.32	1 (Ref.)	1 (Ref.)
Yes	1,183,402	47,442	8,726,217	5.44	2.33 (2.30–2.36)	2.29 (2.26–2.32)
**Reduced HDL C**						
No	3,117,544	72,102	22,902,690	3.15	1 (Ref.)	1 (Ref.)
Yes	451,560	15,956	3,337,963	4.78	1.51 (1.49–1.54)	1.55 (1.52–1.58)
**Elevated BP**						
No	2,295,993	45,256	16,896,105	2.68	1 (Ref.)	1 (Ref.)
Yes	1,273,111	42,802	9,344,548	4.58	1.71 (1.69–1.73)	1.64 (1.62–1.67)
**Elevated glucose**						
No	2,801,650	64,801	20,595,279	3.15	1 (Ref.)	1 (Ref.)
Yes	767,454	23,257	5,645,373	4.12	1.31 (1.29–1.33)	1.25 (1.24–1.27)

^a^Adjusted for age, smoking, alcohol drinking, regular exercise, and income.

PYs, person-years; HR, hazard ratio; CI, confidence interval; aHR, adjusted hazard ratio; TG, triglycerides; HDL C, high-density lipoprotein cholesterol; BP, blood pressure; Ref., reference.

**FIGURE 3:**
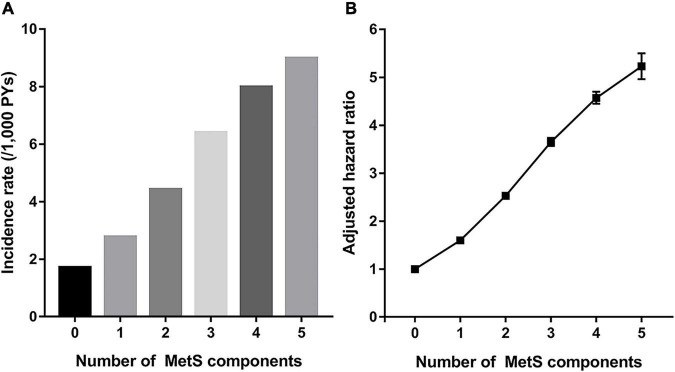
Incidence rate **(A)** and hazard ratio **(B)** for incident gout according to number of components of metabolic syndrome (MetS).

As the number of MetS components increased, the risk of gout increased (Figure 3). Compared with subjects who did not have any MetS components, the subjects with all five MetS components showed a 5-fold increase in risk of gout (aHR 5.24, 95% CI 4.97–5.52). To assess the effect of each MetS component on gout risk, the incidence rates and HRs of gout were analyzed according to positivity of each MetS component (Supplementary Table 1). Compared with subjects who did not have any MetS component, subjects with one or more MetS components showed a higher gout risk. Among subjects with one MetS component, abdominal obesity was associated with the highest risk of gout (aHR 2.33, 95% CI 2.24–2.42), followed by elevated TG (aHR 2.08, 95% CI 2.03–2.14). In subjects with two MetS components, those with both abdominal obesity and elevated TG had the highest risk of gout (aHR 4.03, 95% CI 3.88–4.18).

### Risk factors of gout in young men

Table 3 shows the incidence rates and HRs for gout according to baseline factors in the cohort. Compared with subjects with normal BMI, underweight subjects had half the risk of gout (aHR 0.58, 95% CI 0.53–0.62), and severely obese subjects had a 5-fold increase in gout risk (aHR 5.21, 95% CI 5.09–5.33). As BMI increased, the risk of gout increased dramatically. Heavy drinking was associated with increased risk of gout (aHR 1.82, 95% CI 1.79–1.86). As the TyG index increased, the risk of gout increased.

**TABLE 3:** Hazard ratios and 95% confidence intervals for incident gout according to baseline factors.

	Subjects (*n*)	Events (*n*)	Follow-up duration (PYs)	Incidence rate (per 1,000 PYs)	Crude HR (95% CI)	aHR^a^ (95% CI)
**Age**						
20–29	1,293,621	27,641	9,262,358	2.98	1 (Ref.)	1 (Ref.)
30–39	2,275,483	60,417	16,978,294	3.56	1.17 (1.15–1.19)	1.06 (1.03–1.09)
**Income (quartile)**						
Q1 (lowest)	594,293	12,082	4,210,435	2.87	1 (Ref.)	1 (Ref.)
Q2	1,118,021	26,777	8,085,224	3.31	1.14 (1.12–1.17)	1.11 (1.09–1.13)
Q3	1,213,401	32,215	9,070,581	3.55	1.21 (1.18–1.23)	1.13 (1.11–1.15)
Q4 (highest)	643,389	16,984	4,874,412	3.48	1.18 (1.15–1.21)	1.08 (1.05–1.10)
**BMI (kg/m^2^)**						
<18.5	96,700	660	709,683	0.93	0.56 (0.52–0.60)	0.58 (0.53–0.62)
18.5–23	1,316,908	16,245	9,725,361	1.67	1 (Ref.)	1 (Ref.)
23–25	871,734	18,890	6,441,154	2.93	1.75 (1.72–1.79)	1.72 (1.68–1.75)
25–30	1,093,684	40,293	8,013,762	5.03	3.01 (2.96–3.07)	2.91 (2.86–2.97)
≥30	190,078	11,970	1,350,693	8.86	5.37 (5.24–5.50)	5.21 (5.09–5.33)
**Smoking**						
Never	1,087,843	24,112	7,938,263	3.04	1 (Ref.)	1 (Ref.)
Ex-smoker	528,469	14,693	3,947,029	3.72	1.21 (1.19–1.24)	1.07 (1.05–1.09)
Current smoker	1,952,792	49,253	14,355,360	3.43	1.13 (1.11–1.14)	1.00 (0.99–1.02)
**Alcohol drinking**						
None	942,783	18,389	6,928,610	2.65	1 (Ref.)	1 (Ref.)
Mild (<30 g/day)	2,157,163	52,897	15,875,787	3.33	1.25 (1.23–1.28)	1.24 (1.22–1.27)
Heavy (≥30 g/day)	469,158	16,772	3,436,255	4.88	1.84 (1.80–1.88)	1.82 (1.79–1.86)
**Regular exercise**						
No	3,034,605	73,589	22,298,554	3.30	1 (Ref.)	1 (Ref.)
Yes	534,499	14,469	3,942,098	3.67	1.11 (1.09–1.13)	1.10 (1.08–1.12)
**Comorbidities**						
Hypertension						
No	3,196,701	71,004	23,516,567	3.02	1 (Ref.)	1 (Ref.)
Yes	372,403	17,054	2,724,085	6.26	2.07 (2.04–2.11)	1.96 (1.93–2.00)
**Diabetes mellitus**						
No	3,481,128	85,032	25,594,803	3.32	1 (Ref.)	1 (Ref.)
Yes	87,976	3,026	645,850	4.69	1.41 (1.36–1.46)	1.33 (1.29–1.38)
**Hyperlipidemia**						
No	3,258,150	74,117	23,958,381	3.09	1 (Ref.)	1 (Ref.)
Yes	310,954	13,941	2,282,271	6.11	1.97 (1.94–2.01)	1.90 (1.86–1.93)
**TyG index (quartile)**						
Q1 (lowest)	893,011	10,819	6,511,891	1.66	1 (Ref.)	1 (Ref.)
Q2	891,828	15,626	6,571,072	2.38	1.42 (1.39–1.46)	1.43 (1.39–1.47)
Q3	891,945	23,167	6,588,953	3.52	2.10 (2.05–2.15)	2.11 (2.06–2.16)
Q4 (highest)	892,320	38,446	6,568,737	5.85	3.50 (3.42–3.57)	3.48 (3.41–3.56)

^a^Adjusted for age, smoking, alcohol drinking, regular exercise, and income.

PYs, person-years; HR, hazard ratio; CI, confidence interval; aHR, adjusted hazard ratio; Q, quartile; BMI, body mass index; TyG, triglyceride glucose; Ref., reference.

### Stratified analysis

When stratified analysis was performed based on age, the effect of BMI or MetS on gout was greater in the younger group compared with the older group (Supplementary Table 2). In the presence of MetS, compared with the absence of MetS, the risk of gout was three times higher in subjects with a baseline age of 20–29 years (aHR 3.02, 95% CI 2.94–3.11), and approximately two times higher in subjects with a baseline age of 30–39 years (aHR 2.27, 95% CI 2.23–2.31). There were no significant differences in follow-up duration and time to diagnosis of gout between subgroups according to age.

Supplementary Table 3 presents a stratified analysis according to BMI subgroups. The effect of MetS on gout was greatest in the underweight group (aHR 3.82, 95% CI 2.56–5.72). In particular, in the underweight group, the risk of gout increased 10-fold when abdominal obesity was present (aHR 10.25, 95% CI 5.10–20.61).

## Discussion

In the large nationwide population-based cohort of young men, high BMI, alcohol drinking, and MetS were associated with an increased risk of gout. Compared with subjects without MetS, subjects with MetS had a 2.4-fold increase in the risk of gout. The greater the number of MetS components, the higher the risk of gout. Among the components of MetS, abdominal obesity and elevated TG were most significantly associated with a high risk of gout. The association between gout and MetS was more pronounced in the younger and underweight subjects.

The results of this study demonstrated that MetS and its components were associated with later development of gout. Previous cross-sectional studies have shown associations between MetS and gout or hyperuricemia (15–17, 31, 32). In a prospective study in Taiwan, as in our study, MetS was associated with increased risk of incident gout in men (33). A general population-based longitudinal observational study from Sweden, through cluster analyses, showed that clusters with gout-related comorbidities such as obesity, dyslipidemia, diabetes, and hypertension had a high risk of gout (34). However, the causal interaction between MetS and gout remains unclear. A recent Mendelian randomization study evaluated the causal effect of lipid traits on gout; the results showed that an increase in HDL cholesterol was associated with a decrease in gout risk through a decrease in serum urate, and an increase in TG was associated with an increase in serum urate (35). These findings suggested that MetS and gout may have a causal relationship rather than a simple epiphenomenon. In our study, lifestyle factors were included in the multivariable model to adjust for the influence of lifestyle factors, and even after these variables were adjusted, the association between gout and MetS was significant. In addition, MetS had a greater weight of effect on gout than alcohol drinking, suggesting that the association between MetS and gout was a true correlation, rather than secondary correlation due to shared lifestyle factors. Similarly, in the stratified analysis according to BMI, the association between MetS and gout was significant in all subgroups, although there was a difference in degree of association.

The relationship between MetS and gout is complex, and various mechanisms have been proposed to act as a bridge between the two conditions. Insulin resistance is one of the main players in the pathophysiology of MetS, in which the target tissue exhibits a subnormal coordinated glucose-lowering response at normal plasma insulin levels (36–38). In the state of insulin resistance, insulin secretion is increased to compensate, leading to hyperinsulinemia. Insulin reduces renal excretion of uric acid in the context of euglycemic hyperinsulinemia (39, 40), and recent experimental studies suggested that insulin affects the expression of transporters involved in urate reabsorption (41, 42). In rats, insulin significantly increased the level of urate transporter 1 (URAT1), a major urate reabsorption transporter, and decreased the level of ATP-binding cassette subfamily G member 2 (ABCG2), a major urate secretory transporter, resulting in an increase in uric acid reabsorption (41). In another experimental study using human proximal tubular cells, insulin showed anti-uricosuric effects by promoting the expression and transport activity of glucose transporter 9 (GLUT9), which moves uric acid reabsorbed from the proximal tubule into the blood (42). Therefore, insulin resistance and hyperinsulinemia in MetS can affect the development of gout. In this study, subjects with MetS had a higher TyG index, a surrogate marker of insulin resistance, and higher incidence of gout than subjects without MetS. In addition, as the baseline TyG index increased, the risk of future gout increased.

Among MetS components, abdominal obesity and elevated TG were closely related to the increased risk of incident gout. This result is in line with the previous studies presented on the relationship between visceral obesity and gout. A case-control study comparing visceral fat obesity measured by the bioelectrical impedance analysis method between gout patients and healthy controls showed a significant correlation with an odds ratio of 2.5 between gout and visceral fat obesity (43). Another cross-sectional study using magnetic resonance imaging indicated that hyperuricemia was strongly associated with visceral and hepatic fats, but not subcutaneous fat (44). Visceral adipose tissue has been suggested to contribute to insulin resistance through various mechanisms such as supply of free fatty acid, changes in adipokine secretion, and inflammation (45), and can contribute to gout development. In addition, recent studies have suggested evidence that human adipose tissue secretes hypoxanthine, which can be used as a substrate for uric acid production through xanthine oxidoreductase in other tissues such as liver (45, 46). TG releases free fatty acid by lipoprotein lipase (47). Joosten et al. (48) suggested that free fatty acids may contribute to induction of inflammation in gout by engaging Toll-like receptor 2 and acting synergistically with monosodium urate crystals to activate the apoptosis-associated speck-like protein containing a CARD (ASC)/caspase 1 pathway and induce the release of interleukin-1β. An epidemiological study also showed that hypertriglyceridemia was an independent risk factor for hyperuricemia (49).

Alcohol drinking is a well-known risk factor for gout and was also associated with an increased risk of gout in our study. A previous meta-analysis reported that the pooled relative risk of gout was 1.98 (95% CI 1.52–2.58) for the highest alcohol drinking compared to the non/occasional alcohol drinking (50). In our study, the aHR of gout for the heavy drinking was 1.82 (95% CI 1.79–1.86) compared to the non-drinking. MetS, abdominal obesity and elevated TG had a greater effect on the risk of gout than alcohol drinking. This observation suggests that the effect of MetS on the risk of gout may be due not only to shared lifestyle factors such as alcohol drinking, but also through other mechanisms such as MetS-related genetic factors.

In subgroup analysis, the effect of MetS on incident gout was greater in younger subjects. This was consistent with the results of a cross-sectional study in which the odds ratio for the association between uric acid and MetS was higher in individuals in their 20s than in individuals in their 30s (51). Individuals who develop MetS at an early age are likely to have genetic susceptibility associated with MetS. Some of genetic factors associated with MetS are the same as those for gout (52, 53). Therefore, it is possible that individuals with early development of MetS have more MetS-related genetic factors, resulting in an additional increase in the risk of gout. Previous studies have suggested that the association between MetS phenotype and cardiovascular disease or mortality differs according to age (54, 55). These findings suggest that MetS has a greater effect on the development and prognosis of various diseases in young people, and management of MetS in young people should be emphasized. Since our study was limited to individuals in their 20s and 30s, future studies are needed to evaluate whether the effects of MetS on gout are different in other age groups and to explore the mechanism.

One of the strengths of this study was that a substantial number of subjects who had MetS despite under- and normal-weight was included in the study. The association between MetS and incident gout was more pronounced in under- and normal-weight subjects. Subjects who had MetS despite under- and normal-weight are more likely to have lifestyle problems such as an extremely sedentary lifestyle and heavy consumption of alcohol. Therefore, such problems might affect the occurrence of gout. Previous studies have demonstrated that normal-weight central obesity was associated with a higher risk of cardiovascular disease and mortality (56, 57). In addition, in a recent cross-sectional study of South Korea based on data from the National Health and Nutrition Examination Survey, non-obese males with MetS had a higher risk of hyperuricemia than obese males without MetS (17). This suggests that obesity, defined simply by BMI, might not fully predict the risk of diseases including gout, and that metabolic derangement plays an important role in the pathogenesis of gout.

Our study has several limitations. First, selection bias might exist because this study included only individuals who participated in the health check-up. Second, the incidence of gout might have been over-estimated because gout was defined only by the diagnostic code registered in the claim. Few studies have addressed the incidence of gout in young men. In USA population-based Olmsted county study, the gout incidence in men aged 30–39 years was 1.21 per 1,000 population in 2009–2010 (1), and in the systemic analysis of the Global Burden of Disease Study 2017, the global incidence rate estimate of gout in men aged 35–39 years was 1.75 (95% CI 10.4–26.9) per 1,000 population (58). In a study using the NHIS database in South Korea, the incidence of gout in adults aged 30–39 was 2.05 (95% CI 1.34–2.76) in 2015, and the lower incidence was due to the very low incidence of women (59). The difference in incidence of gout according to race or region and the difference in the definition of gout used in the studies might have caused the difference in incidence of gout among studies. In this study, gout was defined as ≥3 outpatient visits with ICD-10 code, and the positive predictive value of this definition in relation with the ARA, Mexico and Netherland classification criteria was >70% in previous validation study (60). Third, possible confounders were included in the multivariable model, but unmeasured confounders such as inflammatory markers and comorbidities might exist. Fourth, nutritional or dietary factors may act as risk factors for gout, but since information on nutrition or diet was not included in the health check-up variables, such variables were not included in the analysis. Fifth, there is a possibility that the cases where gout occurred after the end of the study were not captured because the subjects were young and the follow-up duration was short with a mean of 7.4 years. Sixth, since serum uric acid was not included in the health check-up variable, we could not analyze the association between hyperuricemia and MetS. Finally, since this study was conducted only on Koreans, there might be limitations in the application of the study findings to other races. However, despite these limitations, the current results reveal the association between MetS and occurrence of gout in a large nationwide population-based cohort of young men.

In conclusion, MetS is an important risk factor for the development of gout in young men. In particular, the association between MetS and gout was greater in young and non-obese men, and the management of MetS in young and non-obese men will be important for gout prevention. Future research on the mechanism by which MetS increases the risk of gout is warranted.

## Data availability statement

The datasets presented in this article are not readily available because restrictions apply to the availability of these data, which were used under license for the current study. Data are however available from the authors upon reasonable request and with permission of NHIS. Requests to access the datasets should be directed to https://nhiss.nhis.or.kr/.

## Ethics statement

The studies involving human participants were reviewed and approved by Institutional Review Board of Samsung Medical Center (IRB File No. SMC 2021-10-008). Written informed consent for participation was not required for this study in accordance with the national legislation and the institutional requirements.

## Author contributions

YE and KH: conceptualization. KH and SWL: data curation and formal analysis. YE, KH, and SWL: investigation. KH, SWL, and KK: methodology. HK and JL: project administration and supervision. YE and SWL: visualization. YE, HK, and JL: writing – original draft. KH, SK, SL, KK, H-SC, E-MK, and JL: writing – review and editing. All authors contributed to the article and approved the submitted version.
