# Supplementary material for: Increased risk of incident gout in young men with metabolic syndrome: A nationwide population-based cohort study of 3.5 million men
Source: Front Med (Lausanne). 2022 Nov 14;9:1010391. doi: 10.3389/fmed.2022.1010391 (PMC9704360; doi:10.3389/fmed.2022.1010391)
Supplement: Supplementary file 1 [file Data_Sheet_1.docx]

**Supplementary Table 1**  Risk of gout according to baseline positivity of each component of metabolic syndrome.

| **No. of MetS components** | **MetS components** | | | | | **Subjects  (*n*)** | **Events  (*n*)** | **Follow-up  duration (PYs)** | **Incidence rate (per 1,000 PYs)** | **aHR^a^ (95% CI)** |
| --- | --- | --- | --- | --- | --- | --- | --- | --- | --- | --- |
|  | **Abdominal obesity** | **Elevated TG** | **Reduced HDL C** | **Elevated BP** | **Elevated glucose** |  |  |  |  |  |
| 0 | No | No | No | No | No | 1,209,361 | 15,294 | 8,875,142 | 1.72 | 1 (Ref.) |
| 1 | Yes | No | No | No | No | 96,943 | 2,819 | 700,256 | 4.03 | 2.33 (2.24–2.42) |
| 1 | No | Yes | No | No | No | 296,232 | 7,995 | 2,200,910 | 3.63 | 2.08 (2.03–2.14) |
| 1 | No | No | Yes | No | No | 81,951 | 1,245 | 607,975 | 2.05 | 1.23 (1.17–1.31) |
| 1 | No | No | No | Yes | No | 437,524 | 7,895 | 3,220,107 | 2.45 | 1.39 (1.36–1.43) |
| 1 | No | No | No | No | Yes | 211,087 | 3,131 | 1,560,858 | 2.01 | 1.16 (1.11–1.20) |
| 2 | Yes | Yes | No | No | No | 68,185 | 3,496 | 496,905 | 7.04 | 4.03 (3.88–4.18) |
| 2 | Yes | No | Yes | No | No | 13,464 | 428 | 97,896 | 4.37 | 2.64 (2.39–2.90) |
| 2 | Yes | No | No | Yes | No | 87,014 | 3,481 | 626,556 | 5.56 | 3.16 (3.05–3.28) |
| 2 | Yes | No | No | No | Yes | 25,926 | 827 | 187,983 | 4.40 | 2.53 (2.36–2.72) |
| 2 | No | Yes | Yes | No | No | 86,179 | 2,488 | 643,746 | 3.86 | 2.30 (2.20–2.40) |
| 2 | No | Yes | No | Yes | No | 182,881 | 6,760 | 1,356,162 | 4.98 | 2.80 (2.72–2.88) |
| 2 | No | Yes | No | No | Yes | 89,237 | 2,635 | 661,396 | 3.98 | 2.27 (2.18–2.37) |
| 2 | No | No | Yes | Yes | No | 27,019 | 574 | 200,858 | 2.86 | 1.70 (1.56–1.84) |
| 2 | No | No | Yes | No | Yes | 14,482 | 239 | 107,868 | 2.22 | 1.34 (1.18–1.52) |
| 2 | No | No | No | Yes | Yes | 121,786 | 2,448 | 900,041 | 2.72 | 1.54 (1.48–1.61) |
| 3 | Yes | Yes | Yes | No | No | 29,854 | 1,629 | 218,504 | 7.46 | 4.43 (4.21–4.66) |
| 3 | Yes | Yes | No | Yes | No | 89,051 | 5,848 | 643,540 | 9.09 | 5.11 (4.95–5.26) |
| 3 | Yes | Yes | No | No | Yes | 29,944 | 1,471 | 217,803 | 6.75 | 3.84 (3.64–4.06) |
| 3 | Yes | No | Yes | Yes | No | 10,741 | 465 | 78,035 | 5.96 | 3.53 (3.22–3.87) |
| 3 | Yes | No | Yes | No | Yes | 3,472 | 103 | 25,357 | 4.06 | 2.44 (2.01–2.96) |
| 3 | Yes | No | No | Yes | Yes | 33,659 | 1,316 | 242,510 | 5.43 | 3.08 (2.91–3.26) |
| 3 | No | Yes | Yes | Yes | No | 49,527 | 1,978 | 369,647 | 5.35 | 3.12 (2.98–3.27) |
| 3 | No | Yes | Yes | No | Yes | 26,541 | 813 | 197,784 | 4.11 | 2.44 (2.28–2.62) |
| 3 | No | Yes | No | Yes | Yes | 83,051 | 3,248 | 612,052 | 5.31 | 2.96 (2.85–3.08) |
| 3 | No | No | Yes | Yes | Yes | 7,285 | 177 | 54,190 | 3.27 | 1.94 (1.68–2.25) |
| 4 | Yes | Yes | Yes | Yes | No | 35,724 | 2,406 | 259,041 | 9.29 | 5.43 (5.20–5.66) |
| 4 | Yes | Yes | Yes | No | Yes | 13,135 | 643 | 95,723 | 6.72 | 3.98 (3.68–4.31) |
| 4 | Yes | Yes | No | Yes | Yes | 55,663 | 3,438 | 400,469 | 8.58 | 4.81 (4.63–4.99) |
| 4 | Yes | No | Yes | Yes | Yes | 3,988 | 174 | 28,804 | 6.04 | 3.60 (3.10–4.18) |
| 4 | No | Yes | Yes | Yes | Yes | 23,683 | 1,004 | 175,684 | 5.71 | 3.32 (3.11–3.54) |
| 5 | Yes | Yes | Yes | Yes | Yes | 24,515 | 1,590 | 176,852 | 8.99 | 5.24 (4.97–5.52) |

^a^Adjusted for age, smoking, alcohol drinking, regular exercise, and income.

No., number; MetS, metabolic syndrome; TG, triglycerides; HDL C, high-density lipoprotein cholesterol; BP, blood pressure; PYs, person-years; aHR, adjusted hazard ratio; 95% CI, 95% confidence interval; Ref., reference.

**Supplementary Table 2** Adjusted hazard ratios and 95% confidence intervals for the association between incident gout and various risk factors in men in early adulthood stratified by age.

|  | **Age (years)** | | ***p* for interaction** |
| --- | --- | --- | --- |
|  | **20–29 (*n* = 1,293,621)** | **30–39 (*n* = 2,275,483)** |  |
| Income (quartile) |  |  | 0.1372 |
| Q1 (lowest) | 1 (Ref.) | 1 (Ref.) |  |
| Q2 | 1.10 (1.07–1.13) | 1.10 (1.06–1.13) |  |
| Q3 | 1.08 (1.04–1.12) | 1.13 (1.10–1.16) |  |
| Q4 (highest) | 1.10 (1.04–1.15) | 1.07 (1.04–1.11) |  |
| BMI (kg/m^2^) |  |  | <.0001 |
| <18.5 | 0.54 (0.48–0.61) | 0.61 (0.55–0.68) |  |
| 18.5–23 | 1 (Ref.) | 1 (Ref.) |  |
| 23–25 | 1.82 (1.76–1.89) | 1.64 (1.60–1.68) |  |
| 25–30 | 3.32 (3.22–3.43) | 2.71 (2.65–2.77) |  |
| ≥30 | 6.35 (6.10–6.61) | 4.66 (4.52–4.80) |  |
| Smoking |  |  | 0.0050 |
| Never | 1 (Ref.) | 1 (Ref.) |  |
| Ex-smoker | 1.03 (0.98–1.07) | 1.08 (1.05–1.10) |  |
| Current smoker | 1.02 (0.99–1.04) | 0.99 (0.97–1.01) |  |
| Alcohol drinking |  |  | 0.2174 |
| None | 1 (Ref.) | 1 (Ref.) |  |
| Mild (<30 g/day) | 1.27 (1.23–1.31) | 1.24 (1.21–1.26) |  |
| Heavy (≥30 g/day) | 1.86 (1.79–1.93) | 1.81 (1.77–1.86) |  |
| Regular exercise |  |  | 0.9076 |
| No | 1 (Ref.) | 1 (Ref.) |  |
| Yes | 1.10 (1.07–1.14) | 1.10 (1.07–1.12) |  |
| Comorbidities |  |  |  |
| Hypertension |  |  | <.0001 |
| No | 1 (Ref.) | 1 (Ref.) |  |
| Yes | 2.11 (2.04–2.18) | 1.92 (1.88–1.96) |  |
| Diabetes mellitus |  |  | 0.0799 |
| No | 1 (Ref.) | 1 (Ref.) |  |
| Yes | 1.42 (1.30–1.56) | 1.33 (1.28–1.38) |  |
| Hyperlipidemia |  |  | <.0001 |
| No | 1 (Ref.) | 1 (Ref.) |  |
| Yes | 2.25 (2.16–2.34) | 1.82 (1.80–1.85) |  |
| Metabolic syndrome |  |  | <.0001 |
| No | 1 (Ref.) | 1 (Ref.) |  |
| Yes | 3.02 (2.94–3.11) | 2.27 (2.23–2.31) |  |
| Abdominal obesity |  |  | <.0001 |
| No | 1 (Ref.) | 1 (Ref.) |  |
| Yes | 3.07 (2.99–3.15) | 2.23 (2.19–2.27) |  |
| Elevated TG |  |  | <.0001 |
| No | 1 (Ref.) | 1 (Ref.) |  |
| Yes | 2.41 (2.36–2.47) | 2.23 (2.19–2.27) |  |
| Reduced HDL C |  |  | <.0001 |
| No | 1 (Ref.) | 1 (Ref.) |  |
| Yes | 1.63 (1.58–1.69) | 1.52 (1.49–1.55) |  |
| Elevated BP |  |  | 0.0235 |
| No | 1 (Ref.) | 1 (Ref.) |  |
| Yes | 1.67 (1.63–1.71) | 1.63 (1.60–1.66) |  |
| Elevated glucose |  |  | 0.0681 |
| No | 1 (Ref.) | 1 (Ref.) |  |
| Yes | 1.27 (1.23–1.31) | 1.25 (1.23–1.27) |  |

Adjusted for age, smoking, alcohol drinking, regular exercise, and income.

Q, quartile; BMI, body mass index; TG, triglycerides; HDL C, high-density lipoprotein cholesterol; BP, blood pressure; Ref., reference.

**Supplementary Table 3** Adjusted hazard ratios and 95% confidence intervals for the association between incident gout and various risk factors in men in early adulthood stratified by body mass index.

|  | **Body mass index (kg/m^2^)** | | | | | ***p* for interaction** |
| --- | --- | --- | --- | --- | --- | --- |
|  | **<18.5**  **(*n* = 96,700)** | **18.5–23**  **(*n* = 1,316,908)** | **23–25**  **(*n* = 871,734)** | **25–30**  **(*n* = 1,093,684)** | **≥30**  **(*n* = 190,078)** |  |
| Income (quartile) |  |  |  |  |  | <0.0001 |
| Q1 (lowest) | 1 (Ref.) | 1 (Ref.) | 1 (Ref.) | 1 (Ref.) | 1 (Ref.) |  |
| Q2 | 1.25 (1.01–1.55) | 1.10 (1.05–1.16) | 1.10 (1.05–1.15) | 1.06 (1.02–1.09) | 1.07 (1.01–1.13) |  |
| Q3 | 1.05 (0.83–1.32) | 1.13 (1.07–1.18) | 1.09 (1.04–1.15) | 1.05 (1.01–1.08) | 1.06 (1.00–1.12) |  |
| Q4 (highest) | 1.20 (0.90–1.59) | 1.11 (1.05–1.17) | 1.04 (0.98–1.09) | 1.00 (0.96–1.04) | 1.02 (0.95–1.09) |  |
| Smoking |  |  |  |  |  | <0.0001 |
| Never | 1 (Ref.) | 1 (Ref.) | 1 (Ref.) | 1 (Ref.) | 1 (Ref.) |  |
| Ex-smoker | 1.14 (0.87–1.50) | 1.11 (1.06–1.17) | 1.08 (1.03–1.13) | 1.01 (0.98–1.05) | 1.01 (0.96–1.07) |  |
| Current smoker | 0.82 (0.69–0.99) | 1.05 (1.01–1.09) | 1.05 (1.02–1.09) | 0.96 (0.94–0.98) | 0.87 (0.83–0.91) |  |
| Alcohol drinking |  |  |  |  |  | <0.0001 |
| None | 1 (Ref.) | 1 (Ref.) | 1 (Ref.) | 1 (Ref.) | 1 (Ref.) |  |
| Mild (<30 g/day) | 1.13 (0.95–1.35) | 1.22 (1.17–1.27) | 1.29 (1.24–1.34) | 1.22 (1.19–1.25) | 1.16 (1.11–1.22) |  |
| Heavy (≥30 g/day) | 1.49 (1.13–1.96) | 1.82 (1.73–1.92) | 1.72 (1.64–1.81) | 1.65 (1.59–1.70) | 1.36 (1.29–1.44) |  |
| Regular exercise |  |  |  |  |  | 0.4592 |
| No | 1 (Ref.) | 1 (Ref.) | 1 (Ref.) | 1 (Ref.) | 1 (Ref.) |  |
| Yes | 0.94 (0.72–1.24) | 1.03 (0.99–1.08) | 1.05 (1.01–1.09) | 1.05 (1.03–1.08) | 1.08 (1.03–1.13) |  |
| Comorbidities |  |  |  |  |  |  |
| Hypertension |  |  |  |  |  | <0.0001 |
| No | 1 (Ref.) | 1 (Ref.) | 1 (Ref.) | 1 (Ref.) | 1 (Ref.) |  |
| Yes | 2.04 (1.49–2.78) | 1.64 (1.55–1.73) | 1.54 (1.47–1.60) | 1.48 (1.45–1.52) | 1.30 (1.25–1.35) |  |
| Diabetes mellitus |  |  |  |  |  | <0.0001 |
| No | 1 (Ref.) | 1 (Ref.) | 1 (Ref.) | 1 (Ref.) | 1 (Ref.) |  |
| Yes | 3.09 (2.12–4.49) | 1.63 (1.47–1.80) | 1.20 (1.09–1.31) | 1.03 (0.98–1.09) | 0.84 (0.78–0.91) |  |
| Hyperlipidemia |  |  |  |  |  | <0.0001 |
| No | 1 (Ref.) | 1 (Ref.) | 1 (Ref.) | 1 (Ref.) | 1 (Ref.) |  |
| Yes | 2.06 (1.35–3.16) | 1.73 (1.63–1.84) | 1.53 (1.47–1.60) | 1.48 (1.44–1.52) | 1.35 (1.30–1.41) |  |
| Metabolic syndrome |  |  |  |  |  | <0.0001 |
| No | 1 (Ref.) | 1 (Ref.) | 1 (Ref.) | 1 (Ref.) | 1 (Ref.) |  |
| Yes | 3.82 (2.56–5.72) | 1.84 (1.72–1.96) | 1.59 (1.52–1.66) | 1.59 (1.56–1.63) | 1.42 (1.36–1.47) |  |
| Abdominal obesity |  |  |  |  |  | <0.0001 |
| No | 1 (Ref.) | 1 (Ref.) | 1 (Ref.) | 1 (Ref.) | 1 (Ref.) |  |
| Yes | 10.25 (5.10–20.61) | 1.52 (1.24–1.85) | 1.33 (1.25–1.42) | 1.36 (1.33–1.39) | 1.18 (1.10–1.27) |  |
| Elevated TG |  |  |  |  |  | <0.0001 |
| No | 1 (Ref.) | 1 (Ref.) | 1 (Ref.) | 1 (Ref.) | 1 (Ref.) |  |
| Yes | 1.75 (1.40–2.19) | 1.81 (1.75–1.88) | 1.74 (1.69–1.79) | 1.75 (1.71–1.78) | 1.60 (1.54–1.67) |  |
| Reduced HDL C |  |  |  |  |  | 0.0004 |
| No | 1 (Ref.) | 1 (Ref.) | 1 (Ref.) | 1 (Ref.) | 1 (Ref.) |  |
| Yes | 1.74 (1.30–2.33) | 1.30 (1.23–1.37) | 1.23 (1.18–1.29) | 1.23 (1.20–1.26) | 1.17 (1.12–1.21) |  |
| Elevated BP |  |  |  |  |  | <0.0001 |
| No | 1 (Ref.) | 1 (Ref.) | 1 (Ref.) | 1 (Ref.) | 1 (Ref.) |  |
| Yes | 1.14 (0.94–1.38) | 1.30 (1.26–1.34) | 1.29 (1.25–1.33) | 1.31 (1.29–1.34) | 1.19 (1.14–1.24) |  |
| Elevated glucose |  |  |  |  |  | <0.0001 |
| No | 1 (Ref.) | 1 (Ref.) | 1 (Ref.) | 1 (Ref.) | 1 (Ref.) |  |
| Yes | 1.40 (1.16–1.70) | 1.15 (1.11–1.20) | 1.11 (1.08–1.15) | 1.10 (1.07–1.12) | 0.98 (0.94–1.01) |  |

Adjusted for age, smoking, alcohol drinking, regular exercise, and income.

Q, quartile; BMI, body mass index; TG, triglycerides; HDL C, high-density lipoprotein cholesterol; BP, blood pressure; Ref., reference.


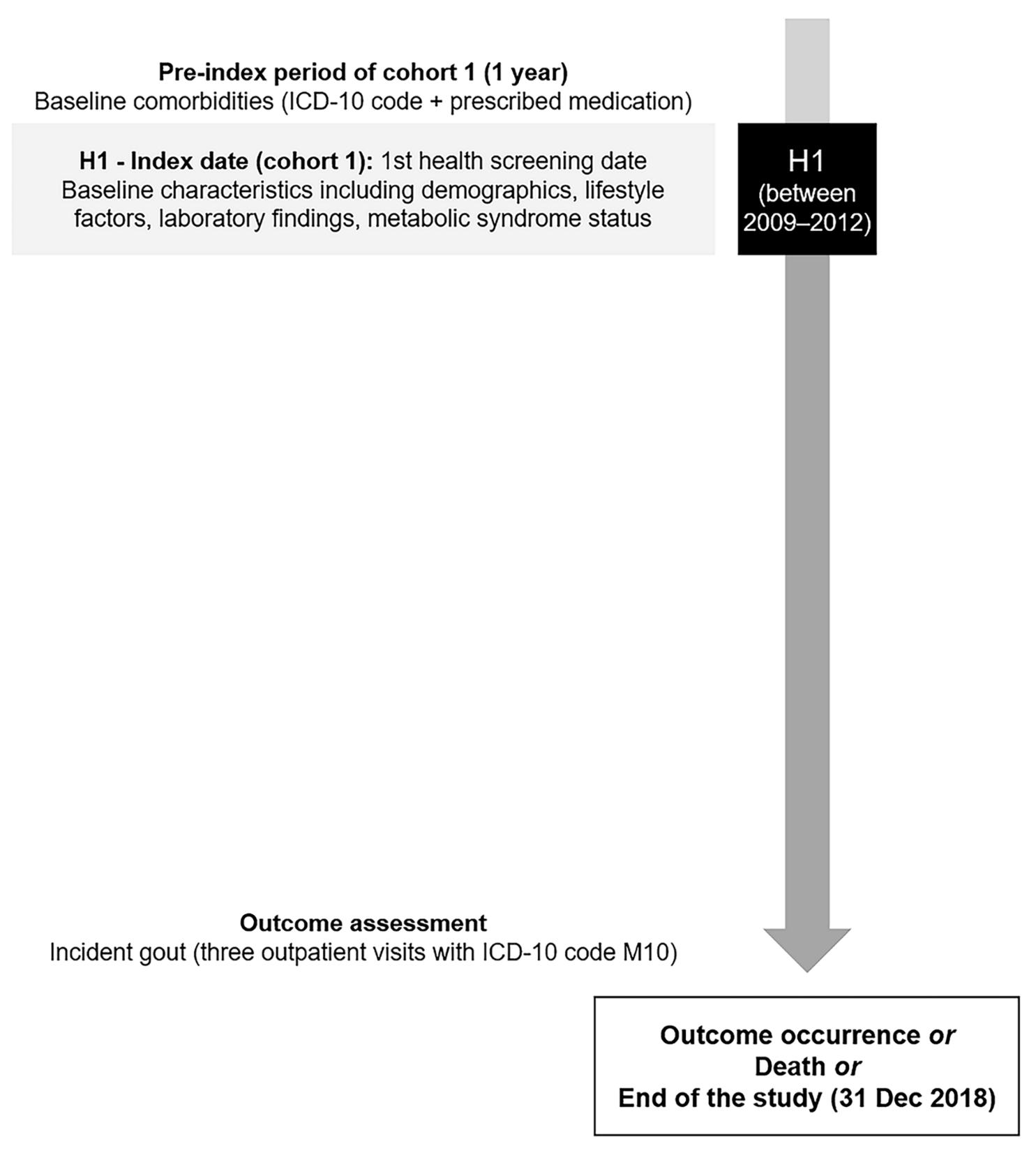


**Supplementary Figure 1**

Study design.
